# Quantitative and O_2_ Enhanced MRI of the Pathologic Lung: Findings in Emphysema, Fibrosis, and Cystic Fibrosis

**DOI:** 10.1155/2007/23624

**Published:** 2007-04-22

**Authors:** Alfred Stadler, Leopold Stiebellehner, Peter M. Jakob, Johannes F. T. Arnold, Edith Eisenhuber, Isabella von Katzler, Alexander A. Bankier

**Affiliations:** ^1^Department of Radiology, Medical University of Vienna, Waehringer Guertel 18-20, 1090 Vienna, Austria; ^2^Department of Internal Medicine IV, Medical University of Vienna, Waehringer Guertel 18-20, 1090 Vienna, Austria; ^3^Institute of Physics, Experimental Physics 5, University of Wuerzburg, 97074 Wuerzburg, Germany; ^4^Department of Radiology, Otto Wagner Hospital, 1140 Vienna, Austria

## Abstract

*Purpose*: beyond the pure morphological visual representation, MR imaging offers the possibility to quantify parameters in the healthy, as well as, in pathologic lung parenchyma. Gas exchange is the primary function of the lung and the transport of oxygen plays a key role in pulmonary physiology and pathophysiology. The purpose of this review is to present a short overview of the relaxation mechanisms of the lung and the current technical concepts of T1 mapping and methods of oxygen enhanced MR imaging. *Material and Methods*: molecular oxygen has weak paramagnetic properties so that an increase in oxygen concentration results in shortening of the T1 relaxation time and thus to an increase of the signal intensity in T1 weighted images. A possible way to gain deeper insights into the relaxation mechanisms of the lung is the calculation of parameter Maps. T1 Maps based on a snapshot FLASH sequence obtained during the inhalation of various oxygen concentrations provide data for the creation of the so-called oxygen transfer function (OTF), assigning a measurement for local oxygen transfer. T1 weighted single shot TSE sequences also permit expression of the signal changing effects associated with the inhalation of pure oxygen. *Results*: the average of the mean T1 values over the entire lung in inspiration amounts to 1199 +/− 117 milliseconds, the average of the mean T1 values in expiration was 1333 +/− 167 milliseconds. T1 Maps of patients with emphysema and lung fibrosis show fundamentally different behavior patterns. Oxygen enhanced MRT is able to demonstrate reduced diffusion capacity and diminished oxygen transport in patients with emphysema and cystic fibrosis. *Discussion*: results published in literature indicate that T1 mapping and oxygen enhanced MR imaging are promising new methods in functional imaging of the lung and when evaluated in conjunction with the pure morphological images can provide additional valuable information.

## 1. INTRODUCTION

Beyond the pure morphological visual representation, MR imaging offers the possibility to quantify functional parameters in healthy, as well as, the pathologic lung tissue. The T1 time of biological tissue is one of the potential measurable parameters. Gas exchange is the primary function of the lung and oxygen uptake plays a major role in lung physiology and pathophysiology. Consequently, the representation and quantification of lung oxygen uptake provides important information regarding pulmonary function. Furthermore, image creation of pathologically altered oxygen exchange in the diseased lung is of great preclinical and clinical interest. Due to the paramagnetic properties of oxygen, MR imaging of oxygen transport is theoretically possible. The purpose of this review is to present a short overview of the relaxation mechanisms of the lung and the current technical concepts of T1 mapping and methods of oxygen enhanced MR imaging.

## 2. PULMONARY RELAXATION MECHANISMS

The first order approximation of the lung T1 relaxation time shows it to be monoexponential and considerably dependant upon tissue water content: the greater the water content the shorter the T1 time [1, 2]. In a majority of studies, alteration of T1 relaxation times in pathologically-changed lungs (i.e., pulmonary edema) could be qualitatively explained by the effect of water in the lungs [3], even though the measurements showed variable quantitative results [4]. The T1 alterations observed in animal experiments, or in ex vivo observations of various pathologies (i.e., Lung fibrosis) could not solely be explained by the effect of lung water [5–8].

The explanation of MR relaxation mechanisms in the presence of macromolecules, such as collagen, was simplified via the development of a theoretical model which is based on the interaction between water molecules and macromolecules [9]. This takes into account that the pulmonary T1 relaxation properties are determined by two balanced groups of water molecules: one compartment consists of free water and the other by water which is bound to macromolecules such as collagen. An alteration of the lung parenchyma, such as the increase in the quantity of macromolecules, leads to a change in the water-bound fraction and therefore a change in the proportion of water-bound molecules.

**Figure 1:**
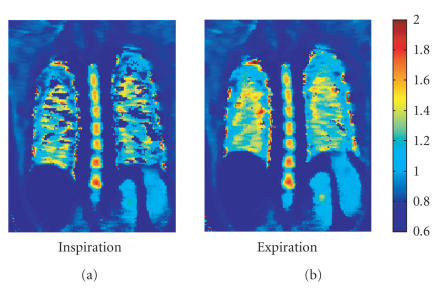
T1 map of a healthy subject: expiration (b) demonstrates a lengthening of the T1 times (seconds) as compared to inspiration (a).

A significant percentage of signal generating protons are found in the blood. Parenchymal regional perfusion will, therefore, have an effect on the regional lung parenchymal T1 relaxation time. Lung perfusion and airspace oxygen content are dependent from the respiratory phase. However, in a recent publication, we illustrated that the T1 time in inspiration (1199 +/− 117 milliseconds) is shorter than in expiration (1333 +/− 167 milliseconds) [10] (Figure 1).

## 3. OXYGEN AS CONTRAST MEDIUM

**Figure 2:**
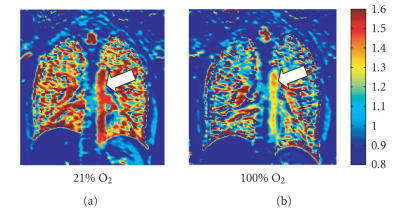
T1 map (seconds) of a healthy subject with inhalation of room air 21% oxygen (a), as well as inhalation of 100% oxygen (b). The increase of O_2_ concentration shortens the T1 time of the lung parenchyma as well as the Aorta (arrow).

Due to two unpaired electrons, molecular oxygen has weak paramagnetic properties with a magnetic moment of 2.8 Bohr magnetons. The idea to use oxygen as a paramagnetic contrast medium is not new and was initially discussed by Young et al. [11]. The potential advantages of oxygen as a contrast medium are obvious. Oxygen is inexpensive, readily available, and safe. Short-term inhalation is not associated with any adverse side-effects. Only after continuous inhalation of 100% oxygen for more than 24 hours does the possibility of permanent pathological lung changes arise [12]. The illustration of oxygen dispersion is of great physiological and pathophysiological interest in that oxygen transport represents the essential function of the lungs and not the distribution of nonphysiological substances which are measured in examinations such as a nuclear medicine-pulmonary function test. A portion of oxygen is bound to hemoglobin in the pulmonary capillary bed and a small fraction remains in soluble form. The hemoglobin bound oxygen is enclosed within the erythrocyte and therefore, the tissue water protons cannot engage in a spin-lattice interaction which leads to T1 relaxation [13]. Edelman et al. were the first to discuss the use of the paramagnetic properties of soluble oxygen to illustrate pulmonary oxygen transport [14]. Inhalation of pure oxygen increases the PaO_2_ in the lungs. This increase in partial oxygen pressure leads to a shortening of the T1 time (Figure 2) and therefore, to a signal rise of the T1 weighted images [15]. Animal studies demonstrated a linear correlation between PaO_2_ and lung parenchymal relaxivity (=1/T1) [16]. The difference in lung parenchymal acquisition signal intensities between inhaled room air and 100% oxygen is minimal, and visual representation is generally accomplished by image subtraction. Apart from the lungs, signal increases were also noted in other organs such as the aorta, spleen, and kidneys [15]. Besides the effect on T1, an elevated oxygen concentration also leads to a prolongation of lung parenchymal T2* time with only a minimal influence on the signal intensity [16].

The exact mechanism altering signal or the change in T1 time is not known. The influence of oxygen on T1 time seems to be played out on the pulmonary vein and parenchymal levels [17]. The molecular oxygen paramagnetic effect is in any case not measurable within the pulmonary gaseous spaces in that the oxygen only influences relaxation of water protons in its proximate surroundings and is itself not signal emitting. After inhalation of higher oxygen concentrations, the interplay of inhalation, diffusion, and perfusion influence the conventional acquisition signal and also alter the T1 time. It only follows that there is a measurable differences in signal behavior in the pathological lung after inhalation of oxygen which results from an alteration of one or more of these factor, thereby increasing interpretation difficulty.

Ohno et al. [18] used the absolute sum of the signal rise during a dynamic measurement as a parameter for lung diffusion capacity, Müller et al. [19] the slope of the rise.

Oxygen Transfer Function (OTF) was created to describe the oxygen transport T1 maps [20]. This was achieved by measuring T1 maps at various inspired oxygen concentrations. Increase in relaxivity is a measure for oxygen transfer of inhaled air into the blood stream. OTF describes the interplay between oxygen diffusion, ventilation, and perfusion.

## 4. TECHNIQUE

MRT examination of the pulmonary parenchyma exhibits a minor signal-to-noise ratio. On the one hand, this is due to minimal lung parenchymal proton density, and on the other, the multiple air/parenchymal surfaces cause susceptibility jumps which results in an extremely short T2 time of only a few milliseconds [21–23]. To achieve the highest possible signal, a short echo time (TE) is necessary. Therefore, the use of a single shot turbo spin-echo (TSE) technique has several advantages: a short TE, multiple 180° refocusing pulses to minimize susceptibility artifacts, a short inter echo time to minimize diffusion and perfusion effects, and a short acquisition time to reduce motion artifacts. In several studies [16, 17, 24, 25] a T1 weighted inversion recovery half-Fourier acquisition single shot turbo spin echo (HASTE) for oxygen enhanced pulmonary MRT was used.

The calculation of T1 maps allows for the elimination of T2 effects and the analysis of the effects of oxygen inhalation. In vivo T1 lung measurements are, as all other MR pulmonary parenchymal measurements, difficult. On the one side, low-proton densities cause a poor signal-to-noise ratio, and conversely, measurements are influenced by susceptibility artifacts as well as motion artifacts (cardiac pulsation and diaphragmatic motion). Most of the published studies examined an only small volume measurement that naturally covers only a minor portion of the lung. This results not only in problems of a statistical nature, but also varying T1 values are expected where it is then unclear if the entire pulmonary parenchyma is represented. In order to evaluate diffuse, but regionally inhomogeneous alterations in lung parenchyma, T1 maps of the entire lung are necessary where there is a corresponding T1 value for every pixel.

**Figure 3:**
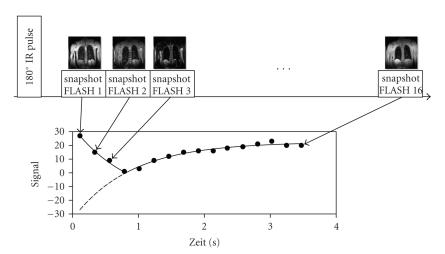
T1 Measurements: after a 180° inversion pulse, 16 Images were acquired at specific time points using a snapshot FLASH sequence. The individual measurements have a duration of 224 milliseconds, the complete slice 3,5 seconds. Due to the short acquisition time, patients with moderate dyspnea can be examined using this method. An exponential function was assigned to each Pixel covering the time span of the 16 measured signal intensities from which a Pixel T1 time can be calculated. The final T1 map results from the color coding of the T1 values.

A possibility which is proposed in several projects, including work from our group, calculates T1 maps based on measurements from snapshot FLASH sequences [10, 26, 27]. These are based on the TOMROP sequence and consist of two elements: first, magnetization is inverted using a nonselective inversion pulse, then the return to original magnetization state occurs over longitudinal relaxation and an image is created using a series of measurements taken from a fast snapshot FLASH sequence [28]. The scan time for one slice at a specific point in time has an estimated duration of 200 milliseconds and is measured at 16 predefined points in time after the inversion pulse. Acquisition of an entire slice lasts about four seconds. The measurement provides 16 time-dependant signal intensities for each pixel. An exponential-fit over these data points yields the T1 relaxation time for each pixel. A color coded image of the T1 values for all the pixels produces the final T1 map (Figure 3).

## 5. EMPHYSEMA

**Figure 4:**
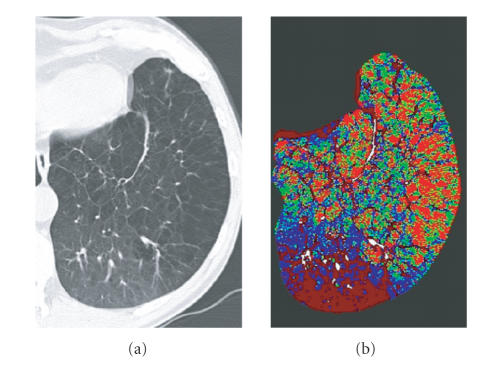
Morphological CT (a) and perfusion map of an emphysematous lung show good correlation of severe emphysema in the anterior-lateral parts of the lung and decreased perfusion (green pixels) as compared to the normal posterior parts of the lung. The bright red pixels represent excluded, air containing parts of the lung.

**Figure 5:**
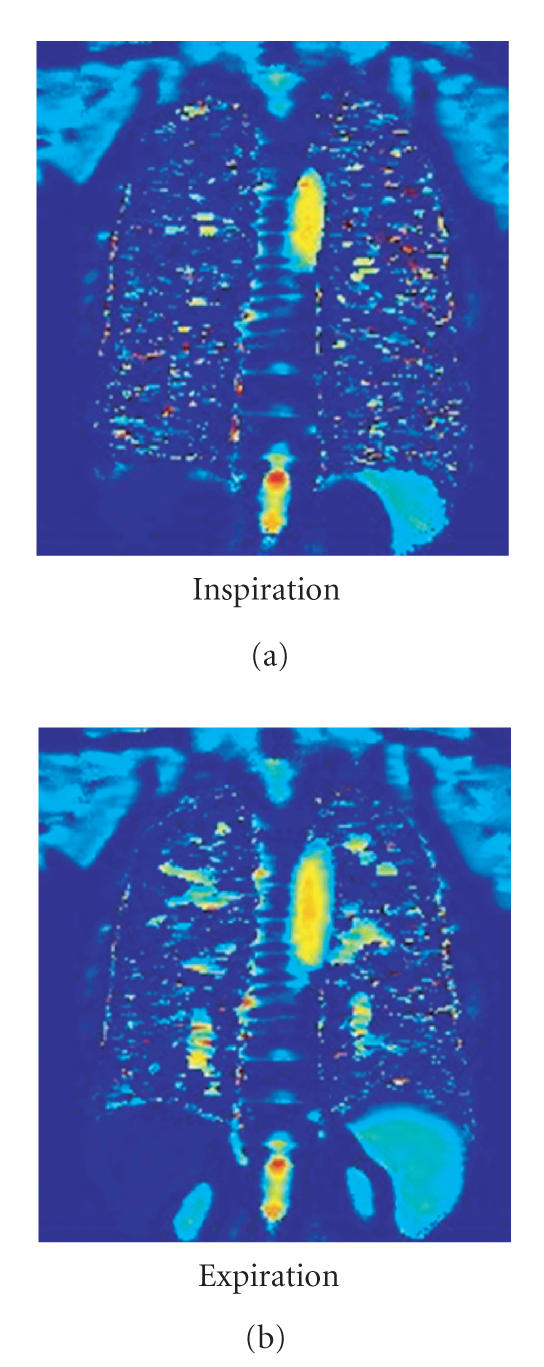
T1 map of a patient with lung emphysema: when compared to a healthy lung (Ill. 1), the T1 time is clearly shortened. Expiration (b) demonstrates no significant prolongation of the T1 time as compared with inspiration (a).

WHO defines emphysema as irreversible enlargement of the air spaces distal to the terminal bronchiole with destruction of the elastic scaffolding without accompanying fibrosis [29]. The detection and the morphological grading of lung emphysema using computer tomography have been intensively explored over the last few years [30]. Furthermore, there are ongoing studies to evaluate the capacity of CT to include functional information like perfusion (Figure 4). The inherent problems associated with pulmonary MR examinations, especially the low-proton density (and the poor signal-to-noise ratio) or the susceptibility artifacts are even more pronounced in the emphysema altered lung. Independent of emphysema pathogenesis, the total composition of macromolecules such as collagen and elastin are within normal limits, however the distribution and the organization are pathologically altered [31]. Signal behavior of the emphysemic lung is, apart from a total reduction in signal intensity which mirrors the pulmonary parenchymal destruction, difficult to foresee. It was recently demonstrated that the T1 relaxation time in emphysematous altered lung is significantly shorter than in healthy lung (Figure 5) [32]. The cause of this T1 time shortening possibly lies in vascular rarefaction or a redistribution of blood within the effected lung parenchyma. In any case, this has to be taken into consideration when performing inhalation examinations. At this time, there is no definitive data available relating to T1 relaxation time behavior after 100% oxygen inspiration.

A study from Ohno et al. compared healthy volunteers with emphysema patients [18], and healthy volunteers with patients diagnosed with bronchial carcinoma and Bronchial carcinoma patients without emphysema to those with [25], respectively. The time course of an acquisition was examined by taking sequential measurements using HASTE sequencing with inhalation of 21% (room air) and 100% oxygen.

The 100% oxygen signal rise in patients with pulmonary emphysema was significantly flatter and demonstrated excellent correlation with FEV1. Strong correlation between the maximum signal rise and CT emphysema scoring as well as pulmonary diffusion capacity was also noted. Müller was also able to show a reduction in diffusion capacity in the emphysemic lung segments when compared to healthy subjects through dynamic measurements of pulmonary signal behavior during inhalation of 100% oxygen. Contrary Ohno's results, it was demonstrated that inspiration of 100% oxygen provided a good correlation between the slope of the signal rise and the clinical measurement of diffusion capacity.

The existing studies show that oxygen enhanced pulmonary MRT can be a valuable complementary tool in evaluating pulmonary emphysema patients, especially in spatial encoded imaging of pulmonary diffusion capacity. Investigations in this field are, however, still in the preclinical trial phase.

## 6. FIBROSIS

**Figure 6:**
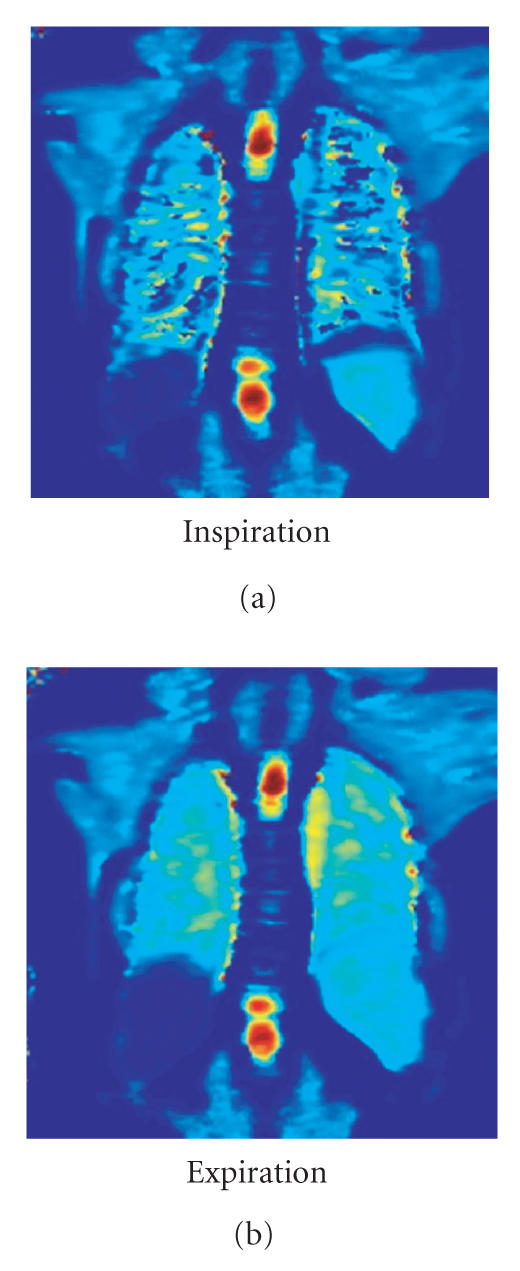
T1 map of a Patient with lung fibroses: in comparison to a healthy lung (Figure 2), the T1 time is shortened. Expiration (b) shows a significant T1 time prolongation as compared to inspiration (a).

Independent of the etiology, pulmonary fibrosis is characterized by the deposition of newly-synthesized matrix molecules. In accordance with the above described simplified two compartment model, the relative increase in macromolecules should lead to a shortening of the T1 relaxation time. This assumption was partially verified by animal model measurements [7], and in some measurements there was no change in T1 time noted [6]. Our measurements demonstrated that patients with lung fibrosis, when compared to healthy volunteer subjects, showed a shortening in the T1 relaxation time which is less pronounced in expiration than in inspiration (Figure 6) [32]. However, there are no current publications discussing the behavior of the fibrotic lung under 100% oxygen inspiration.

## 7. CYSTIC FIBROSIS

Cystic fibrosis is an autosomal-recessive hereditary disease in which the pathological composition of the exocrine gland secretion leads to characteristic secondary changes in target organs. In the lungs, this disease has a homogeneous picture encompassing atelectasis, emphysema, microabscesses, bronchiectasis which ultimately leads to pulmonary fibrosis.

**Figure 7:**
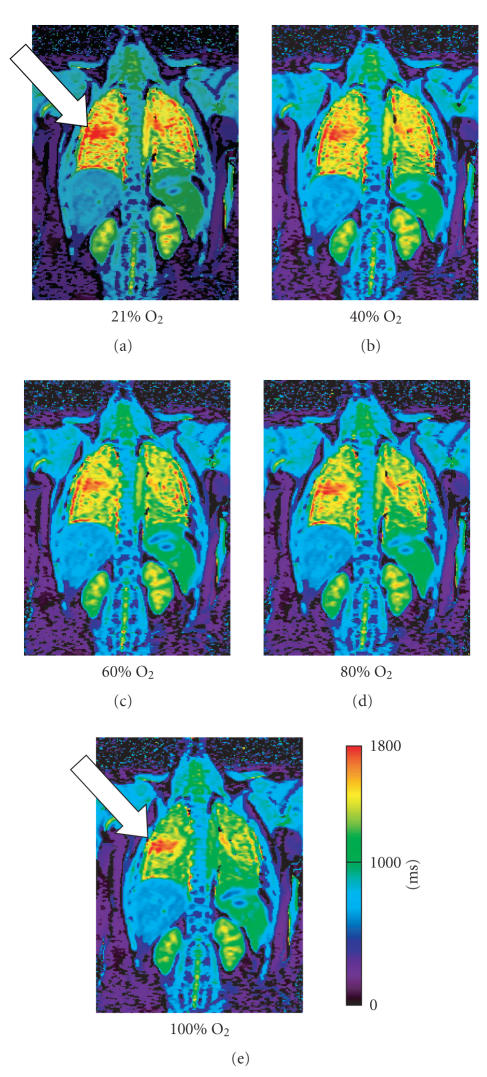
T1 map (millisecond) of a patient with Cystic Fibrosis: inhalation of increasing oxygen concentrations demonstrates a steady shortening of T1 times in the healthy lung segments. The diseased tissue of the right mid lobe (arrow) distinguishes itself in that the T1 time remains relatively unchanged.

**Figure 8:**
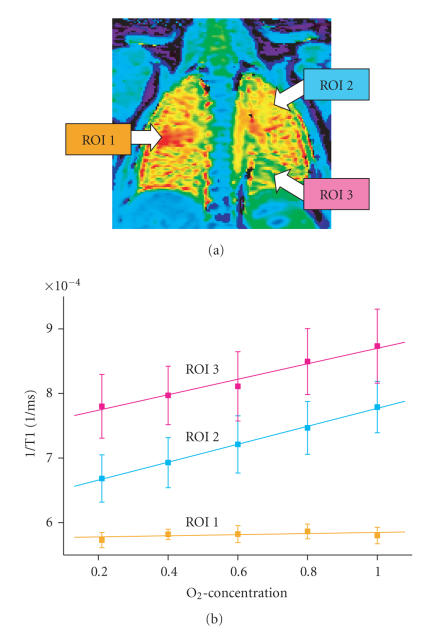
Oxygen transfer function (OTF) of a patient with Cystic Fibroses from Ill. 6. Healthy lung segments (ROI 2 and 3) demonstrate a linear increase in relaxivity (= 1/T1) with increasing oxygen concentrations. The slope of the curve is a measurement of lung oxygen transport capacity. The diseased lung segment (ROI 1) shows no rise; there is therefore no oxygen transport.

In a recent work, T1 maps of healthy volunteers were compared to those of cystic fibrosis patients [20]. The patient group showed an inhomogeneous distribution of T1 relaxation times where pathologically altered segments had shorter T1 times than the noneffected segments as well as the lungs of the healthy subjects (Figure 7). The OTF curve of the pathological lung segments exhibited a distinctly flattend pattern, consequently, a reduced dependence of pulmonary parenchymal relaxivity on the inhaled oxygen concentration (Figure 8). Reasons for this change can either be related to the limited pulmonary diffusion capacity or the alteration in ventilation or perfusion of the diseased segments, thus a supplementary MR pulmonary perfusion study is recommended.

## 8. SUMMARY

T1 maps from healthy subjects and patients with emphysema and fibrosis reveal significantly different behaviors. These differences reflect the complex interaction of the structural and functional influences of the above-mentioned diseases. Pulmonary relaxation mechanisms are still not fully understood. Further studies using T1 maps can advance the understanding of the relationship between lung structure and lung function. A basic knowledge of the T1 relaxation mechanisms is also the ground work for optimizing conventional MR pulmonary image sequencing. All published data, up to now, confirm that oxygen enhanced MRT has an important role in the imaging of diffusion capacity and oxygen transport which provides valuable information in the detection and understanding of the role that functional alterations have in lung diseases such as emphysema and cystic fibrosis.

In spite of the considerable technical difficulties, several publications confirm the potential that T1 maps and oxygen enhanced MRT have characterizing pathological changes in lung tissue. However, existing literature still cannot provide a final evaluation of the presented methods. The previously obtained results will allow for further informative insight into the functional changes of the pathological altered lung parenchyma.
